# The *Neurospora crassa* TOB Complex: Analysis of the Topology and Function of Tob38 and Tob37

**DOI:** 10.1371/journal.pone.0025650

**Published:** 2011-09-28

**Authors:** Sebastian W. K. Lackey, Jeremy G. Wideman, Erin K. Kennedy, Nancy E. Go, Frank E. Nargang

**Affiliations:** Department of Biological Sciences, University of Alberta, Edmonton, Alberta, Canada; Consejo Superior de Investigaciones Cientificas, Spain

## Abstract

The TOB or SAM complex is responsible for assembling several proteins into the mitochondrial outer membrane, including all β-barrel proteins. We have identified several forms of the complex in *Neurospora crassa*. One form contains Tob55, Tob38, and Tob37; another contains these three subunits plus the Mdm10 protein; while additional complexes contain only Tob55. As previously shown for Tob55, both Tob37 and Tob38 are essential for viability of the organism. Mitochondria deficient in Tob37 or Tob38 have reduced ability to assemble β-barrel proteins. The function of two hydrophobic domains in the C-terminal region of the Tob37 protein was investigated. Mutant Tob37 proteins lacking either or both of these regions are able to restore viability to cells lacking the protein. One of the domains was found to anchor the protein to the outer mitochondrial membrane but was not necessary for targeting or association of the protein with mitochondria. Examination of the import properties of mitochondria containing Tob37 with deletions of the hydrophobic domains reveals that the topology of Tob37 may be important for interactions between specific classes of β-barrel precursors and the TOB complex.

## Introduction

The vast majority of proteins found in mitochondria are encoded by nuclear genes and synthesized in the cytosol. These proteins must be imported into mitochondria, directed to the proper mitochondrial subcompartment, and properly assembled at their final destination. The mitochondrial outer membrane houses the TOM complex (translocase of the outer membrane) which recognizes most mitochondrial precursor proteins in the cytosol and imports them into the organelle. A few outer membrane proteins partition directly into the outer membrane via the TOM complex or other assembly routes. However, the majority of mitochondrial precursor proteins are transported through the membrane where targeting information within the precursors enable further interactions with specific factors and/or complexes in the intermembrane space and/or the mitochondrial membranes. These interactions lead to the completion of accurate subcompartment targeting for the incoming precursor proteins [1], [2], [3], [4].

β–barrel proteins form a specific class of mitochondrial protein that exist exclusively in the outer membrane of the organelle. They are imported through the TOM complex to the intermembrane space where they are chaperoned to the inner surface of the outer membrane by the small Tim protein complexes [5], [6] for interaction with the TOB complex (topogenesis of β-barrel proteins), also known as the SAM complex (sorting and assembly machinery). The TOB complex assembles the β-barrel precursor proteins into the outer membrane [1], [2], [3], [7], [8]. The known β-barrel proteins of the outer mitochondrial membrane include: Tom40, Tob55 (Sam50, Omp85), porin (VDAC), Mdm10, and possibly Mmm2. In addition to its role in the assembly of β-barrel proteins, the *Saccharomyces cerevisiae* TOB complex has been shown to be involved in the integration and/or assembly of several TOM complex proteins that are anchored in the membrane via C-terminal α-helical domains (discussed below).

A core TOB complex has been defined in *S. cerevisiae* [9], [10], [11] that contains Tob55, Tob38 (Sam35, Tom38), and Tob37 (Tom37, Sam37, Mas37). (To minimize confusion we will henceforth use the names Tob55, Tob38, and Tob37 to refer to the subunits of the fungal TOB core complex.) Recent studies suggest that the core complex has the capacity to partner with several other proteins resulting in the formation of dynamic complexes with specialized function. For example, the core complex is known to associate with the Mdm10 protein in *S. cerevisiae* [12], [13], [14], [15] and *Neurospora crassa* [16]. Mdm10 is a multifunctional protein that was originally described for its role in mitochondrial distribution and morphology [17]. It was found to exist in a complex with Mdm12 and Mmm1, which are also involved in maintenance of mitochondrial morphology [18]. Subsequently the three proteins were shown to be part of a mitochondrial/ER tethering system known as the ERMES (endoplasmic reticulum mitochondria encounter structure) complex [19]. Binding of Mdm10 to the TOB complex has been shown to play a role in Tom40 assembly [12], [13], [14], [15]. However, the exact role of Mdm10 is currently unresolved. One model suggests that Mdm10 stimulates release of Tom40 precursor from the TOB complex [15]. Another model suggests that the requirement of Mdm10 for Tom40 assembly is indirect because integration of Tom22 into the membrane requires the action of the TOB complex associated with Mdm10 [14] and Tom22 is necessary for assembly of Tom40 into the TOM complex [20], [21]. Recently, an additional form of the TOB complex containing endogenous Tom5 and Tom40 proteins (referred to as SAM-Tom5/Tom40) was described in *S. cerevisiae* [14]. Co-purification experiments have also shown an interaction between the TOB complex and the Mim1 (Tom13) protein [22] and a genetic interaction between Tob37 and Mim1 has also been demonstrated [23].

The function and topology of the individual components of the TOB core complex have been investigated to varying degrees. Tob55 is essential for viability in *S. cerevisiae* and *N. crassa* [24], [25], [26], [27]. The protein is itself a β-barrel protein and is thought to form a pore that enables incoming precursors to enter the membrane [24], [28]. Homologues of Tob55 have been identified in organisms as diverse as mammals and gram-negative bacteria [24], [25], [26]. Tob55 contains a polypeptide transport-associated (POTRA) domain that plays a role in releasing β-barrel precursor proteins from the TOB complex [29]. Tob38 of *S. cerevisiae* is also an essential protein but is found as a peripheral membrane protein on the cytosolic side of the outer membrane [9], [10], [11]. Despite its topology, the protein has been shown to interact with precursor proteins that have entered the intermembrane space. This interaction occurs between the β-signal, found at the C-terminus of β-barrel precursor proteins, and a domain of Tob38 that most likely becomes available to the β-signal via membrane embedded protein-protein interactions with Tob55 [28].

Early investigations into the role of Tob37 in *S. cerevisiae* demonstrated that it was not essential for viability, but cells lacking the protein had growth defects at high temperature [30]. Originally, the protein was thought to interact with Tom70 as an import receptor for mitochondrial precursors that lacked matrix targeting signals, such as AAC (ATP/ADP carrier) [30]. Subsequently, it was shown that Tob37 did not act as a receptor for AAC import [31] and that the protein was actually part of the TOB complex [32]. *S. cerevisiae* Tob37 is thought to be a peripheral membrane protein because it can be removed from the mitochondrial outer membrane by alkali extraction [31]. Both Tob37 and Tob38 interact with Tob55 in *S. cerevisiae*. This interaction is likely responsible for binding the two peripheral membrane proteins to the membrane [9], [10], [11], [25], [28]. Tob37 is thought to act later in the process of assembling β-barrels by assisting release of substrates from the TOB complex [33], [34]. Interestingly, a close genetic relationship between Tob37 and Tom6 has been described in which overexpression of one protein suppresses defects seen when the other protein is absent. Deletion of both genes was found to be synthetically lethal. It was suggested that since Tom6 plays a role in stabilizing Tom40, overexpression of Tom6 could compensate for the decreased assembly of Tom40 that resulted from absence of Tob37 [34]. Overexpression of Mdm10, Mdm12, or Mmm1 has also been shown to partially suppress the growth phenotype of cells lacking Tob37 [35]. Recently, Tob37 has been shown to be involved in a complex series of pathways involving phospholipid metabolism, endoplasmic reticulum-mitochondrial interactions, and cell wall integrity in *Candida albicans* [36].

Mammalian mitochondria contain Tob55 [37] as well as weakly conserved homologues of Tob38 and Tob37, known as Metaxin 2 (Mtx2) and Metaxin 1 (Mtx1), respectively [10], [38], [39]. The components and size of the mammalian TOB complex are presently unclear. In one study [37], Tob55 was found in a complex of about 200 kDa by BNGE. Antibodies against Tob55 shifted an import complex containing a stalled Tom40 precursor to a higher molecular weight. However, antibodies against Mtx1 did not shift the complex. In another study [40], two-dimensional gel electrophoresis revealed that Tob55 was present in a complex of more than 200 kDa, but Mtx2 was found in a 600 kDa complex. Most of Mtx1 was present as a low molecular weight species, but also appeared in a smear from 200 kDa to over 600 kDa [40], [41]. It was concluded that Mtx1 and Mtx2 are present in a complex separate from the Tob55 containing complex [40]. A different study showed all three proteins to be a part of a larger complex, along with several other proteins, that was immunoprecipitated from human heart mitochondria using antibody to mitofilin [42].

Mtx1 contains a signal anchor sequence near its C-terminus and the protein is not removed from the outer membrane by alkali extraction [38], [39]. Removing the signal anchor sequence severely reduced targeting and association of the protein with mitochondria [38]. Mtx1 was found to be essential for mouse development [43]). Mtx2 is considered to be a peripheral outer membrane protein based on alkali extraction experiments. Since Mtx1 and Mtx2 have been shown to physically interact, it was suggested that Mtx1 binds Mtx2 to the membrane [39]. Early studies suggested that Mtx1 had an effect on the import of precursors destined for the mitochondrial matrix [38], [41]. A later study concluded that mitochondria depleted of Mtx2 were deficient in the assembly of β-barrel proteins and that the import of matrix destined precursors was not affected [40].

Thus, despite several recent advances, many questions regarding the nature and function of the TOB complex and its components remain controversial. Differences in the topology, function, and place of Tob37 in the TOB complex between the *S. cerevisae* protein and mammalian Mtx1 are particularly evident. Here we describe an investigation into the *N. crassa* TOB complex. We have examined the nature of the complex and the properties of Tob37 and Tob38 mutants. The existence of two possible membrane spanning hydrophobic regions near the C-terminus of the *N. crassa* Tob37 protein suggested that in this fungal species the protein more closely resembled its mammalian orthologue in its structure. Thus, to gain further insight into the function of Tob37 and its properties in different species, we investigated the roles of these hydrophobic domains in the *N. crassa* protein.

## Methods

### Ethics statement

All work with animals used in the production of antibodies was conducted according to the guidelines established by the Canadian Council on Animal Care. Antibodies against Tob37, Tob38, and Mdm10 were raised in guinea pigs and mice for this study and were described previously [16]. Methods for injection of antigens and removal of blood were approved by the Biological Sciences Animal Policy and Welfare Committee of the University of Alberta, protocol number 587.

### Strains and growth of *N. crassa*

Strains used in this study are listed in Table 1. *N. crassa* was grown according to previously described procedures [44]. Unless otherwise stated, cells were grown at 30°C. Tests of growth rate were performed as described previously [27].

**Table 1:** Strains used in this study.

Strain (short name)	Genotype	Origin or reference
76–26	*his-3 mtrR a* (*mtrR* imparts fpa resistance)	R.L. Metzenberg
71–18	*pan-2 BmlR a* (*BmlR* imparts benomyl resistance)	R.L. Metzenberg
HP1	Heterokaryon of 76-26 plus 71–18.	Nargang Lab. [46]
Tob37KO-5 (ΔTob37)	Sheltered heterokaryon. As HP1, but with replacement of *tob37* gene in 76–26 nucleus with a hygromycin resistance (*hygR*) cassette.	Transformation of HP1 with split marker fragments for *tob37* knockout.
Tob38KO-6 (ΔTob38)	Sheltered heterokaryon. As HP1, but with replacement of *tob38* gene in 76–26 nucleus with a hygromycin resistance (*hygR*) cassette.	Transformation of HP1 with split marker fragments for *tob38* knockout.
Tob37HT (9His-Tob37-2)	*his-3 mtrR a Δtob37::hygR* contains an ectopic copy of genomic *tob37* with C-terminal 9x His tag. Bleomycin resistant.	Nargang Lab [16]
Tob38HT (9His-Tob38-3)	*his-3 mtrR a Δtob38::hygR* contains an ectopic copy of genomic *tob38* with C-terminal 9x His tag. Bleomycin resistant.	Nargang Lab [16]
Tob55HT (H6C4–5)	*his-3 mtrR a Δtob55::hygR* contains an ectopic copy of genomic *tob37* with N-terminal 9x His tag. Bleomycin resistant.	Nargang Lab [16]
Tob55 Short HT	*his-3 mtrR a Δtob55::hygR* contains an ectopic copy of N-terminal 9x His tagged *tob55* cDNA specific for the short form.	Nargang Lab
Tob55 Int HT	*his-3 mtrR a Δtob55::hygR* contains an ectopic copy of N-terminal 9x His tagged *tob55* cDNA specific for the intermediate form.	Nargang Lab
Tob55 Long HT	*his-3 mtrR a Δtob55::hygR* contains an ectopic copy of N-terminal 9x His tagged *tob55* cDNA specific for the long form.	Nargang Lab
Tob37ΔTMD1–9	*his-3 mtrR a Δtob37::hygR* contains an ectopic copy of genomic *tob37* ***Δ*** *TMD1*. Bleomycin resistant.	Nargang Lab
Tob37ΔCHD2–3	*his-3 mtrR a Δtob37::hygR* contains an ectopic copy of genomic *tob37* ***Δ*** *CHD*. Bleomycin resistant.	Nargang Lab
Tob37ΔT+C12–5	*his-3 mtrR a Δtob37::hygR* contains an ectopic copy of genomic *tob37* ***Δ*** *T+C*. Bleomycin resistant.	Nargang Lab

### Construction of Tob37 and Tob38 knockout strains

A split marker approach was used to knock out the *tob37* and *tob38* genes. Approximately three kilobase regions upstream and downstream of the coding sequence for each gene were generated via PCR of cosmids containing the genes, or from genomic DNA. These regions and a hygromycin resistance cassette were used in the construction of the appropriate split markers [45] for each gene as described previously for *N. crassa tob55* [27]. For each gene, the two portions of the split marker were transformed into heterokaryon HP1 [46]. Hygromycin resistant colonies were isolated, purified, and examined for replacement of the *tob37* or *tob38* gene in one of the nuclei of HP1 by Southern analysis (not shown). Strains showing the correct pattern of integration were then examined for growth characteristics. One nucleus of the heterokaryon carries an allele (*mtr*) for resistance to p-fluorophenylalanine (fpa) plus auxotrophy for histidine, while the second carries benomyl resistance (Bml) and pantothenate auxotrophy. Transformation of *N. crassa* typically occurs in only one nucleus of a multi-nucleate conidium [47]. To determine which nucleus of the heterokaryotic transformants was transformed by the split marker and carried the knockout, the strains were tested for their ability to grow on medium containing either histidine plus fpa or pantothenate plus benomyl. For isolates with the knockout in the histidine-requiring, fpa-resistant nucleus, the presence of fpa in the growth medium forces the nucleus containing the knockout to predominate the culture, resulting in a deficiency of Tob37 or Tob38. If the proteins are required for maximal growth rate, such knockouts should grow slowly under these conditions. One strain showing this phenotype for each gene was chosen for further analysis: for Tob37, strain Tob37KO-5; for Tob38, strain Tob38KO-6.

To obtain mitochondria with reduced levels of Tob37 or Tob38, liquid cultures of knockout heterokaryons were grown in the presence of histidine plus fpa for 36 to 40 hours, compared to 16 to 20 hours for cultures without histidine and fpa. Controls were grown for about 20–24 hours in histidine plus fpa, or 16–20 hr without histidine and fpa.

### Creation of strains carrying altered versions of Tob37

Mutant alleles of *tob37* were created by site-directed PCR mutagenesis of a Bluescript plasmid containing the genomic copy of *N. crassa Tob37* and a bleomycin resistance gene [48]. Mutagenesis was performed to create *Hpa*I restriction sites flanking the regions containing two possible transmembrane domains (TMDs). Following mutagenesis, coding regions between residues 386–405, 434–452, and 386–452 could be removed by *Hpa*I digestion and religation. Because of the coding capacity of the restriction sites, the deletions are flanked with Val (GTT) and Asn (AAC) codons. Plasmids confirmed to contain the desired mutations by sequence analysis were linearized and used to transform conidia from the sheltered heterokaryon strain Tob37KO-5. The transformation mixture was plated on medium containing histidine, and fpa to select for the nucleus bearing the *tob37* knockout, as well as bleomycin to select for transformants carrying the plasmid. Transformants were purified through one round of single colony isolation on medium containing fpa, histidine, and bleomycin. Colonies were picked and tested for nutritional requirements. Transformants that required histidine were homokaryons that contained the desired mutant alleles, which must be capable of restoring Tob37 function to a level sufficient for viability. The presence of the correct mutant alleles in the transformants was confirmed by sequencing PCR products of the ectopically integrated *tob37* mutant alleles from isolated genomic DNA.

### Salt treatment of isolated mitochondria

Mitochondria (50 µg protein), were suspended in 50 µl of isolation buffer (0.25 M sucrose, 1 mM EDTA, 10 mM MOPS, pH 7.2) containing 1 mM phenlymethylsulfonyl fluoride (PMSF)), plus 500 mM NaCl and left on ice for 30 min. The sample was then centrifuged at 16,250 x *g* at 4°C for 20 min in a refrigerated microcentrifuge. The mitochondrial pellets were processed for electrophoresis by dissolving in cracking buffer (0.06 M Tris-HCl, pH 6.8; 2.5% SDS; 5% β-mercaptoethanol; 5% sucrose). The supernatant was desalted using the Zeba™ Spin Desalting Column system (Pierce Biotechnology – Thermo Scientific, Rockford, IL) and then prepared for electrophoresis by adding one fifth volume of 5X cracking buffer.

### Gel electrophoresis of proteins

Proteins were analyzed by sodium dodecyl sulphate polyacrylamide gel electrophoresis (SDS-PAGE) or BNGE as described previously [49], [50], [51] using 30 µg or 50 µg of mitochondrial protein per lane, respectively, unless stated otherwise. For two dimensional electrophoresis, lanes from the first dimension BNGE were excised and soaked in cracking buffer for 5 min. Treated lanes were then placed between the glass gel plates, onto the stacking gel, of a pre-made gel for second dimension SDS-PAGE. Following electrophoresis, gels were transferred to either nitrocellulose or PVDF (polyvinylidene fluoride) membrane and immunodecorated with specific antibodies.

### Electrophoretic analysis of affinity purified proteins

Affinity purification of His-tagged proteins was performed as described [16]. For BNGE of purified His-tagged proteins, one tenth volume of 10X sample buffer (100 mM bis-Tris, pH 7.0; 500 mM 6-aminocaproic acid; 5% Coomassie brilliant blue G250) was added directly to the elution fractions. These were immediately subjected to BN-PAGE. After electrophoresis gels were transferred to PVDF for Western analysis.

### Proteinase K treatment of Isolated Mitochondria

Isolated mitochondria (50 µg) were suspended in 150 µl of isolation buffer on ice. Following addition of proteinase K (8 µl from a stock solution of 2 mg/ml) the sample was incubated for 15 min on ice. The proteinase K was then inactivated by the addition of 1.5 µl PMSF (200 mM in ethanol) and an additional 340 µl of isolation buffer containing 1 mM PMSF. After gentle mixing the sample was then centrifuged at 16,250 x *g* at 4°C for 20 min in a microcentrifuge. The mitochondrial pellets were dissolved in 50 ul of cracking buffer and subjected to SDS-PAGE.

### Standard procedures

Mitochondria were isolated as described previously [16], [52]. Alkali extraction was performed as described [16] except that different pHs of sodium carbonate were used in this study as stated for individual experiments. Preparation of damaged mitochondria [16], import of mitochondrial precursor proteins [53], and transformation of *N. crassa* [27] were performed as described previously. Irrelevant lanes were sometimes removed electronically from blots or gels for the production of figures. Comparative quantification of bands on x-ray film was performed on scanned images using Adobe Photoshop.

## Results

### Development of sheltered heterokaryons harbouring nuclei with knockouts of *tob37* or *tob38*

**Figure 1:**
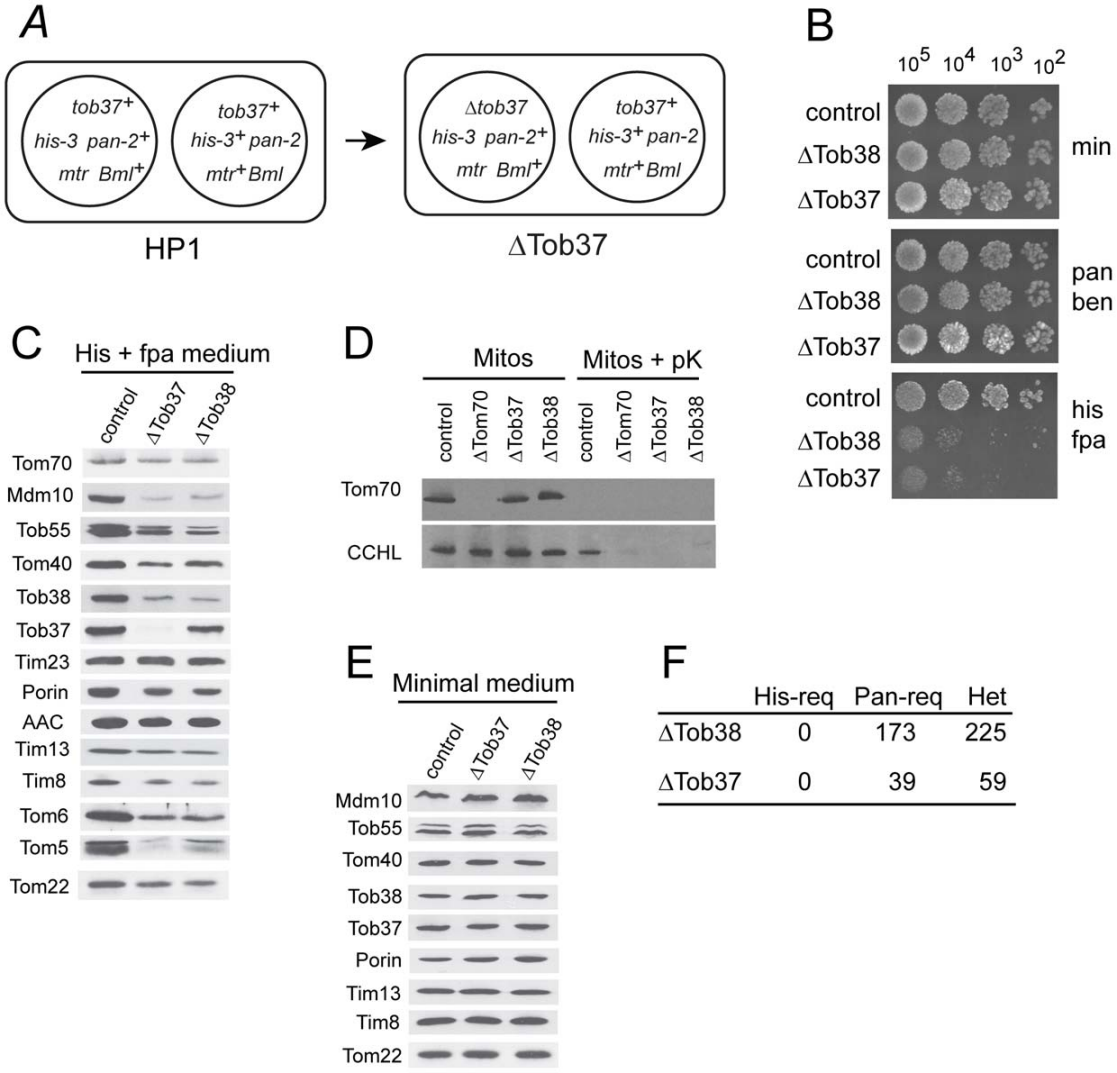
Isolation and characterization of *N. crassa* strains with reduced levels of Tob37 or Tob38. (a) Sheltered heterokaryons with deletions of either *tob37* or *tob38* in one nucleus of the heterokaryon were constructed using a split marker approach. Boxes symbolize heterokaryons while circles within the boxes represent the different component nuclei of the heterokaryon. The Figure shows an example for *tob37*, but the process was identical for *tob38*. The starting heterokaryon (HP1) contained nuclei with different genetic markers: either *his-3* and *mtr* (provides resistance to fpa) or *pan-2* and *Bml* (provides resistance to benomyl). Strains chosen for further work carried the knockouts in the *his-3 mtr* nucleus (see Methods). (b) Serial dilutions of conidiaspores (actual numbers spotted shown at top of panel) from the strains indicated on the left were spotted onto plates containing either minimal medium (min), which maintains both nuclei of the heterokaryon approximately equally; minimal medium containing pantothenate and benomyl (pan ben), which forces the nucleus carrying benomyl resistance (*Bml*, Figure 1A) to predominate the culture; or minimal medium containing histidine plus fpa (his fpa), which forces the nucleus carrying fpa resistance (*mtr*, Figure 1A) to predominate the culture. The control was strain HP1. (c) Cells from the indicated strains (top of panel) were grown in the presence of histidine (His) and fpa to force the predominance of the nucleus bearing the deletion of either *tob37* or *tob38*. This results in reduction of the levels of Tob37 or Tob38, respectively. Mitochondria were isolated and subjected to SDS-PAGE followed by transfer to nitrocellulose, and immunodecoration with the anitbodies indicated on the left. The control strain was HP1. Multiple bands in the Tob55 lane correspond to different isoforms of the protein [27]. (d) Mitochondria isolated from the indicated strains were either untreated (Mitos) or incubated in the presence of proteinase K (Mitos + pK) for 15 min. Mitochondrial proteins were then subjected to SDS-PAGE and western blotting. The blot was examined for the presence of Tom70 and the intermembrane space protein CCHL. (e) As in panel C, except strains were grown in minimal medium which maintains the numbers of both types of nuclei in the culture approximately equally. (f) Conidia produced from the sheltered heterokaryons (ΔTob37 and ΔTob38) were streaked onto medium containing histidine and pantothenate. Individual colonies were isolated and tested for nutritional requirements to determine if they were histidine-requiring homokaryons (His-req), pantothenate requiring homokaryons (Pan-req), or heterokaryons (Het).

We generated knockouts of the *N. crassa tob37* and *tob38* genes via transformation of the HP1 heterokaryotic strain using a split marker approach as described previously for *N. crassa tob55* [27]. Transformation of one of the two different nuclei in HP1 results in the creation of a sheltered heterokaryon (Figure 1A). As described in the Methods, we chose one strain for each gene where the knockout was in the histidine-requiring (*his-3*), fpa resistance (*mtr*) nucleus. These sheltered heterokaryon strains will hereafter be referred to as ΔTob37 and ΔTob38. Growth of these strains in the presence of histidine and fpa results in a severely reduced growth rate (Figure 1B) as the knockout-containing nuclei are forced to predominate the culture to supply resistance to fpa thus reducing the levels of Tob37 or Tob38. Mitochondria isolated from these strains grown in the presence of histidine and fpa were examined for the presence of various mitochondrial proteins (Figure 1C). Reduction of Tob37 in ΔTob37 also resulted in a reduction of the level of Tob38 but reduction of Tob38 in ΔTob38 had only a minor effect on Tob37 levels. This agrees with previous suggestions that Tob37 may be involved in the association of Tob38 with mitochondria or its stability. Both sheltered heterokaryons grown in the presence of histidine and fpa show a reduction in all β-barrel proteins examined (Mdm10, Tob55, Tom40, and porin). Tom5, Tom6, and Tom22 of the outer membrane were also reduced. Slight reductions of the intermembrane space proteins Tim8 and Tim13 were observed. Levels of the inner membrane protein Tim23 and the outer membrane protein Tom70 were unaffected. These observations are consistent with a role for the TOB complex in the assembly of β-barrel proteins and certain α-helical anchored proteins of the TOM complex.

The reduction in the intermembrane space proteins Tim8 and Tim13 suggests possible breakage of mitochondrial outer membranes during the isolation of mitochondria as we have observed previously for other mutants affecting mitochondrial outer membrane proteins [16]. We investigated this further by examining isolated mitochondria for the presence of the intermembrane space protein cytochrome *c* heme lyase (CCHL) following exposure to proteinase K. In mitochondria from ΔTob37, ΔTob38, and ΔTom70, a mutant previously shown to have mitochondria that were damaged upon isolation [54], the CCHL was degraded by the proteinase (Figure 1D). This demonstrates increased accessibility of the proteinase to the intermembrane space. It should also be noted that levels of CCHL in untreated mitochondria are similar in all the strains examined, as for Tim23 and Tom70 (Figure 1C,D). When ΔTob37 and ΔTob38 were grown in minimal medium, which forces the strains to grow as heterokaryons with relatively equal contributions from both nuclei due to the complementing auxotrophic mutations, all proteins examined in isolated mitochondria were essentially at wild type levels (Figure 1E). To determine if Tob37 and Tob38 were essential for viability, we examined the nutritional requirements of colonies arising from conidiaspores produced by the ΔTob37 and ΔTob38 strains. No histidine-requiring auxotrophs were found for either strain (Figure 1F). This demonstrates that both Tob37 and Tob38 are essential for viability in *N. crassa*.

### Import/assembly of mitochondrial precursor proteins in mitochondria deficient for Tob37 or Tob38

**Figure 2:**
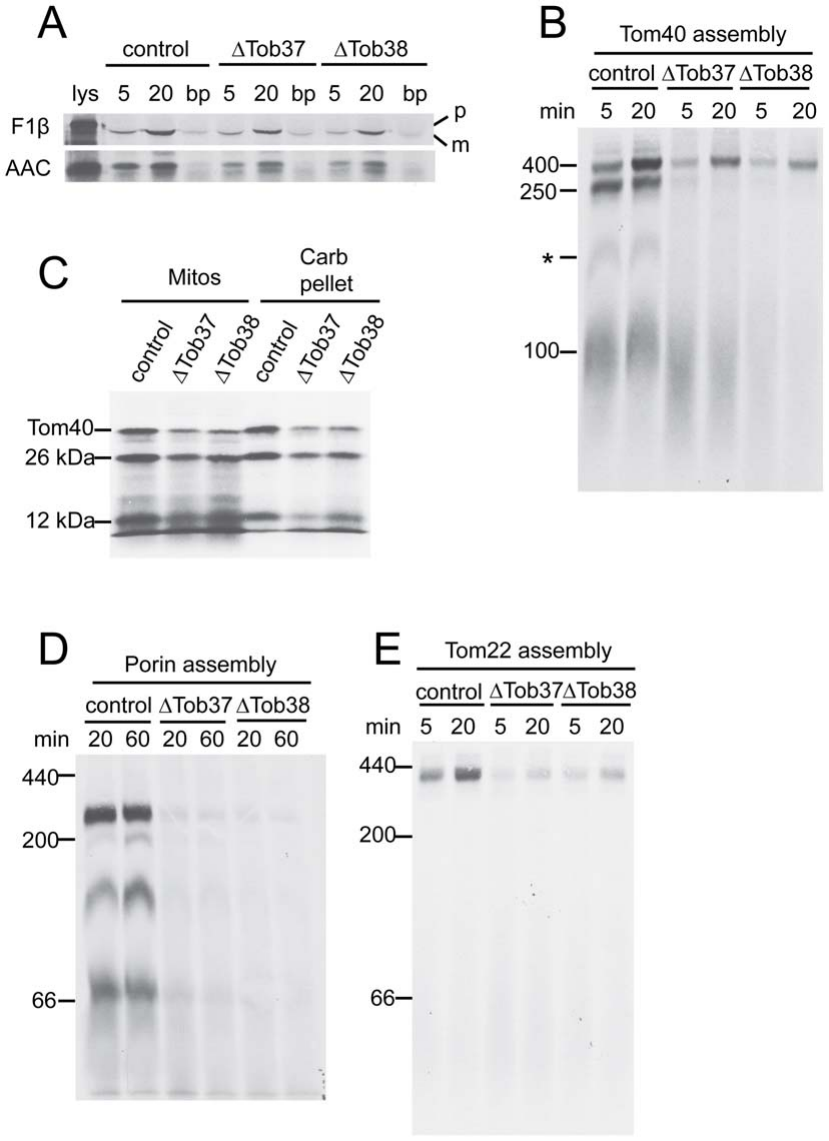
Import/assembly of mitochondrial precursor proteins into mitochondria deficient in Tob37 or Tob38. (a) Radiolabeled matrix precursor F_1_β and inner membrane precursor AAC were incubated (for 5 min or 20 min, as indicated) with mitochondria isolated from heterokaryotic strains (indicated at the top of the panel) grown in the presence of histidine and fpa to reduce levels of Tob37 or Tob38 in the respective mutants. Following import, mitochondria were subjected to SDS-PAGE. Proteins were transferred to nitrocellulose membrane, and import was analyzed by autoradiography. (control, strain HP1; lys, 33% of the radiolabeled lysate added to each import reaction; bp, “bypass import” in mitochondria treated with trypsin to remove surface receptors prior to the import reaction; p, precursor protein; m, mature protein.) (b) Radiolabelled Tom40 precursor was incubated for 5 min and 20 min with mitochondria isolated from the strains indicated (top of panel) grown in the presence of histidine and fpa. Mitochondria were dissolved in 1% digitonin and subjected to BNGE. The proteins were transferred to PVDF membrane and analyzed by autoradiography. The size of the mature TOM complex (400 kDa), and assembly intermediates I (250 kDa) and II (100 kDa) are indicated on the left. * indicates an undefined band. (c) Tom40 was imported into mitochondria isolated from the strains indicated for 20 min. Following import, proteinase K was added to each import reaction for 15 min on ice. PMSF was added to inactivate the proteinase, each reaction was divided into equal halves, and mitochondria were pelleted. One half was suspended in SDS-PAGE cracking buffer (Mitos). The other half was suspended in sodium carbonate (pH 11.5) and incubated on ice for 30 min. The membrane sheets were pelleted and suspended in cracking buffer (Carb pellet). Both sets of reactions were subjected to SDS-PAGE and the proteins were transferred to nitrocellulose membrane and examined by autoradiography. The positions of Tom40 and the 26 kDa and 12 kDa fragments generated by proteinase K digestion are indicated. (d) As in panel B except that mitochondria were incubated with the radiolabeled precursor of porin. The numbers on the left indicate the position of molecular weight markers. (e) Assembly of Tom22. As in panel D, except mitochondria were incubated with radiolabeled Tom22.

Next, we examined import and assembly of mitochondrial precursor proteins in mitochondria isolated from ΔTob37 and ΔTob38 grown in the presence of histidine and fpa to reduce the levels of Tob37 and Tob38, respectively. Import of the matrix targeted precursor F_1_β was found to be slightly reduced to 64% (standard deviation 11%) of the control in ΔTob37 and to 43% (standard deviation 17%) in ΔTob38 mitochondria (Figure 2A). Similarly, import of AAC was slightly reduced to 64% (standard deviation 18%) of the control in ΔTob37 and 47% (standard deviation 10%) in ΔTob38 mitochondria (Figure 2A). Alterations in the assembly of the β-barrel protein Tom40 (Figure 2B) was also observed with both mutants. In wild type mitochondria the Tom40 precursor is imported across the outer membrane and can then be detected associated with the TOB complex as intermediate I of 250 kDa. The precursor is then integrated into the membrane where it forms intermediate II of 100 kDa containing an endogenous Tom40 molecule and a Tom5 subunit. The precursor then proceeds to the fully assembled 400 kDa complex [32], [55], [56], [57]. Particularly striking for Tom40 assembly in mitochondria deficient in Tob37 or Tob38 was the lack of accumulation of the precursor at the 250 kDa first intermediate stage of assembly (Figure 2B), which represents a Tom40 precursor protein at the TOB complex. A substantial amount of Tom40 does seem to reach the final assembled state in the 400 kDa complex in a time dependent fashion. This is similar to the assembly pattern we observed for mitochondria depleted of Tob55 [27]. To insure that the Tom40 observed in the 400 kDa complex was properly assembled, the import was repeated and followed by treatment with proteinase K, which cleaves assembled *N. crassa* Tom40 into 26 kDa and 12 kDa fragments [56]. The ratio of these fragments in the mutants compared to control mitochondria was similar to the ratios observed for the undigested protein in fully assembled TOM complex (compare Figure 2B and Figure 2C). These fragments were also shown to be resistant to alkali extraction (Figure 2C) consistent with the idea that Tom40 had been integrated into the membrane. We conclude that some Tom40 precursor is assembled into mitochondria containing reduced levels of Tob37 and Tob38 and is correctly integrated into the TOM complex. One difference between the ΔTob37 and ΔTob38 mitochondria with respect to Tom40 assembly was the low level of material evident at the 100 kDa position of intermediate II for ΔTob38. The ΔTob37 mitochondria contained a substantial amount of precursor in this region, but the band was more diffuse and lower in molecular weight than in the control mitochondria. These observations demonstrate specificity of function for each protein and imply that Tob37 acts following membrane integration of Tom40 while Tob38 acts in an earlier step. The assembly pattern for porin showed a much reduced efficiency for assembly into all complexes in the mutant mitochondria (Figure 2D). The exact nature of the different porin complexes is not understood, though we have shown that the highest molecular weight complex contains porin precursor bound to the TOB complex [27]. Finally, we demonstrated that the assembly of Tom22 precursor into the TOM complex was also reduced in both mutants (Figure 2E).

The decrease of AAC import (Figure 2A) and the slight deficiency of Tim8 and Tim13 in mitochondria reduced for Tob37 or Tob38 (Figure 1C) was reminiscent of previous observations for mutants lacking the Mdm10 protein [16]. In the latter case, we demonstrated that loss of the small Tim proteins was likely due to breakage of the outer mitochondrial membrane during the isolation of mitochondria from mutant cells. Since the small Tim proteins are known to be involved in the assembly of both AAC [58], [59], [60], [61] and β-barrel proteins [5], [6], [62], we showed that the effects on AAC import, but not the effects on the β-barrels could be explained by the loss of the small Tim proteins from the intermembrane space during the isolation of mitochondria lacking Mdm10 [16]. Similar experiments in this study revealed that control mitochondria, in which the outer membranes had been purposefully damaged to allow loss of intermembrane space components like Tim8 and Tim13, were more severely affected in their ability to import AAC than mitochondria lacking Tob37 or Tob38. However, the damaged control mitochondria were much more efficient at import and assembly of Tom40 and porin than were Tob37 or Tob38 deficient mitochondria (Figure S1). Thus, reductions in the level of Tob37 or Tob38 are the major factor responsible for the deficiencies in the assembly of β-barrel proteins while reduced in vitro import of AAC appears to be at least partly due to the loss of factors from the intermembrane space. However, it should be noted that Tob37 was originally characterized as having an effect on the import of AAC [30]. Since our observations suggest that steady state levels of AAC are also slightly reduced in strains depleted of either Tob37 or Tob38 (Figure 1C) it is conceivable that these proteins also play a minor or indirect role in AAC import/assembly.

### TOB complexes in *N. crassa* mitochondria

**Figure 3:**
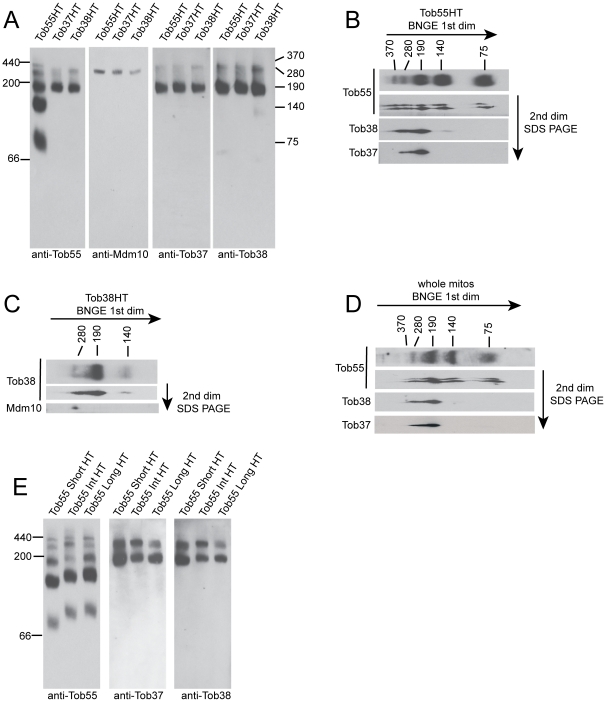
The *N. crassa* TOB complex. (a) Number and protein content of *N. crassa* TOB complexes. Mitochondria were isolated from strains expressing only His-tagged versions (instead of the endogenous versions) of either Tob55 (Tob55HT), Tob38 (Tob38HT), or Tob37 (Tob37HT) as indicated for each lane at the top of the panel. TOB complexes were purified using Ni-NTA resin. Purified complexes were subjected to BNGE, transferred to PVDF membrane, and decorated with antibodies to Tob55, Mdm10, Tob37, or Tob38 as indicated at the bottom of each panel. The position of molecular weight markers (kDa) is shown on the left and the estimated size (kDa) of complexes is shown on the right. (b) TOB complex was purified from a strain carrying His-tagged Tob55 and subjected to first dimension BNGE (1^st^ dim) in two separate lanes of the gel. One lane was transferred to PVDF, and decorated with antibody to Tob55 (top lane in panel). The second lane was removed for second dimension (2^nd^ dim) electrophoresis by SDS-PAGE as described in the Methods. Following SDS-PAGE, the gel was transferred to nitrocellulose. The membrane was cut into strips corresponding to the molecular weights of Tob55, Tob38, and Tob37 and probed with antibodies to those proteins, respectively (indicated on the left). Sizes of TOB complex following 1^st^ dimension BNGE are indicated at the top of the panel. (c) As in panel B, except the purification was performed using mitochondria containing His-tagged Tob38 and the SDS-PAGE blot was examined with antibodies to Tob38 and Mdm10. (d) As in panel B except that whole mitochondria were examined for the presence of TOB complexes. (e) As in panel A, except TOB complex was purified from mitochondria isolated from cells expressing only His-tagged versions of different Tob55 isoforms [27]: short Tob55 (Tob55 Short HT), intermediate Tob55 (Tob55 Int HT), or long Tob55 (Tob55 Long HT) as indicated at the top of the panels. Blots were immunodecorated with the antibodies indicated at the bottom of the panels.

The import data discussed above for strains with reduced levels of Tob37 or Tob38, together with our previous findings for Tob55 [27], demonstrate that the three proteins have similar functions in the import of β-barrel proteins into the outer membrane. We have previously shown that the proteins co-purify as a complex [16], but the number of TOB complexes, their components, and their size has not been investigated in *N. crassa*. BNGE examination of the complex purified from mitochondria containing His-tagged versions of the different TOB core-complex components reveals that Tob55, Tob38, and Tob37 are all found together in two complexes of about 280 kDa and 190 kDa (Figure 3A). In addition, Tob55 appears alone in two smaller complexes of about 75 and 140 kDa and one larger complex of 370 kDa. These latter three complexes are virtually devoid of Tob37 or Tob38, though a small amount of Tob38 is detectable in the 140 kDa form. Two-dimensional gel electrophoresis (BNGE followed by SDS-PAGE) of TOB complex purified from mitochondria containing His-tagged Tob55 confirms these observations (Figure 3B), as does the finding that the 370 kDa, 140 kDa, and 75 kDa complexes are not observed when purification is performed using His-tagged Tob37 or Tob38 (Figure 3A). Mdm10 appears only in the 280 kDa complex (Figure 3A). This was also confirmed by two-dimensional gel analysis (Figure 3C) of TOB complex purified from mitochondria containing His-tagged Tob38. Thus, the 190 and 280 kDa complexes appear to correlate with the TOB core- and holo-complexes, respectively, that have been defined in *S. cerevisiae* [9], [10], [11], [12], [15], [35]. It should be noted that in mock purifications using control mitochondria without His-tagged proteins that no bands correlating with these complexes are observed when blots are immunodecorated with antibodies to Tob55, Tob38, Tob37, or Mdm10 [16].

When whole mitochondria were examined by western blot for Tob55 following BNGE or two-dimensional gel electrophoresis, a pattern similar to that observed for purified complexes was seen (Figure 3D). Thus, it appears unlikely that any of the complexes observed are artefacts of the purification procedure. However, we cannot be certain that all bands detected represent physiologically relevant complexes or if some are breakdown products resulting from BNGE.

*N. crassa* contains three different isoforms of Tob55 [27]. To determine if any of these was specific for a given complex or set of complexes, mitochondria were isolated from strains expressing only the His-tagged versions of either the short, intermediate, or long form of Tob55. TOB complexes were purified and analyzed by BNGE and western blot. In each case, all five TOB complexes described above were present (Figure 3E). In addition, the distribution of Tob37 and Tob38 was similar to cells expressing all three isoforms. We conclude that the different Tob55 isoforms are not involved in the formation of specific TOB complexes.

### Topology of Tob37 and Tob38

**Figure 4:**
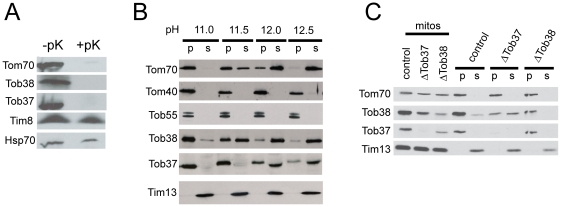
Topology of Tob37 and Tob38. (a) Mitochondria isolated from wild type cells (strain 76-26) were treated with proteinase K (+pK) as in Figure 2C or not (-pK). Mitochondria were washed and subjected to SDS-PAGE. The gel was blotted to nitrocellulose and examined by immunodecoration with the antibodies indicated on the left. (b) Mitochondria from the control strain (76-26) were subjected to extraction with 0.1 M sodium carbonate at the pHs indicated at the top of the panel. Following the treatment, membrane sheets were pelleted and supernatants were subjected to trichloroacetic acid precipitation. Membrane pellets (p) and supernatant precipitates (s) were subjected to SDS-PAGE. Proteins in the gel were transferred to nitrocellulose and the membrane was examined with the antibodies indicated on the left. (c) As in panel B except extractions were done at pH 11.0 from control (HP1), ΔTob37, and ΔTob38 strains grown in the presence of histidine and fpa. The three lanes on the left show the protein levels in whole mitochondria (mitos), while the six lanes on the right show the pellets and supernatants resulting from alkali extraction.

**Figure 5:**
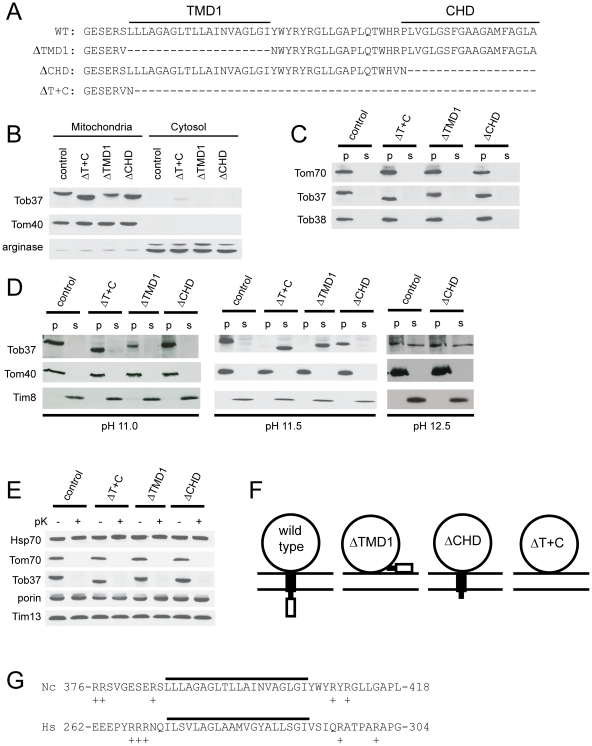
Role of predicted TMDs of Tob37. (a) The WT row shows the sequence of the 63 amino acids at the C-terminus of wild type *N. crassa* Tob37. ΔTMD1 is the deletion constructed for the first possible transmembrane domain and is the name of the strain expressing this form of Tob37. Similarly for CHD, the second possible TMD found at the C-terminus of the protein, and for ΔT+C, the deletion of the last 56 amino acids of the protein which removes both possible TMDs. (b) Mitochondria and post-mitochondrial supernatants (cytosol) were isolated from strains expressing the mutant forms of Tob37 described in panel A. Samples of each were subjected to SDS-PAGE and transferred to nitrocellulose. The membrane was immunodecorated with the antibodies indicated on the left. The control was strain 76-26. Arginase represents a cytosolically localized control protein that is synthesized from two different start codons of the same locus [67] so that two bands of 41 kDa and 36 kDa are observed. (c) As in panel A except isolated mitochondria were treated for 30 min on ice with isolation buffer containing 0.5 M NaCl. Following the incubation period, mitochondria were pelleted. The supernatant was collected and desalted. The mitochondria were washed in isolation buffer and pelleted. Pelleted mitochondria and the desalted supernatant were subjected to SDS-PAGE. Proteins were transferred to nitrocellulose and the membrane was probed with the antibodies indicated on the left. (d) Mitochondria from the strains indicated above the panel were subjected to alkali treatment using 0.1 M sodium carbonate at pH 11.0, 11.5, and 12.5 (indicated below each panel) as described in the legend to Figure 4B. (e) Mitochondria were isolated from each of the Tob37 deletion protein strains and treated with proteinase K as described in the Materials and Methods and Figure 4A. (f) The large circle represents the cytosolic domain of Tob37, the filled box is TMD1, and the open box is the CHD. Two horizontal lines represent the mitochondrial outer membrane. The predicted arrangement of the domains for wild type and each of the TMD/CHD deletions is indicated. (g) Comparison of potential tail-anchoring sequences of *N. crassa* (Nc) Tob37 and *H. sapiens* (Hs) Mtx1. The potential TMD is indicated by the solid line. The position of the region within each protein is indicated by the numbers flanking each amino acid sequence. The overall length of the *N. crassa* protein is 442 residues. The *H. sapiens* protein is 304 residues. Positively charged residues in the immediate flanking regions are indicated by the plus sign.

Both Tob37 and Tob38 are susceptible to degradation by proteinase K added to isolated mitochondria (Figure 4A), showing that the proteins have domains exposed on the outer surface of the outer membrane as does the control protein Tom70. The intermembrane space protein Tim8, and the matrix protein Hsp70 are protected from the externally added protease. We also examined the properties of *N. crassa* Tob37 and Tob38 by alkali extraction at varying pHs. The soluble protein Tim13, appears in the extracted, supernatant phase at all pH levels tested while the β-barrel proteins Tom40 and Tob55 partition with the pelleted membrane sheets (Figure 4B). For Tob38, about half the protein is removed from the membrane at pH 11.5 and the majority is removed at pH 12.0 (Figure 4B). Thus, the behaviour of the protein is similar to the Tom70 protein which is known to have a single membrane spanning domain [63], [64], [65]. However, analysis of the Tob38 amino acid sequence reveals no strong candidates for a membrane spanning helix. Tob37 is more resistant to alkali extraction than Tob38. Very little of the protein is removed from the membrane at pH 11.5, but at pH 12.0 it is roughly equally partitioned between the membrane and supernatant fractions (Figure 4B). Thus, it appears to be a membrane anchored protein as it is slightly more resistant to extraction than Tom70. However, the protein is more easily removed from the membrane than are the β-barrel membrane proteins Tom40 and Tob55. It has been suggested that Tob37 anchors Tob38 to the mitochondrial membrane in mammals [39] or that Tob37 is required for stability of Tob38 in yeast [33], [34]. We examined this possibility by subjecting mitochondria depleted for Tob37 to alkali extraction at pH 11.0. In control mitochondria, Tob38 stays almost entirely associated with the pelleted membrane sheets. However, in the absence of Tob37, the reduced amount of Tob38 that remains in mitochondria is about equally portioned between the pellet and supernatant fractions (Figure 4C). These data support a role for Tob37 in binding Tob38 to mitochondria, but also suggest that Tob38 is bound to the membrane by other interactions. Analysis of the Tob37 amino acid sequence suggests the presence of two possible TMDs (Figure 5A). The first occurs in a position that ends 35 residues before the C-terminus. This placement of the TMD resembles the position of the TMD of mammalian Mtx1 and we refer to the domain as TMD1. The second comprises the last 19 residues of the protein and is referred to as the C-terminal hydrophobic domain (CHD). Taken together, the above data suggest that Tob37 is anchored to the mitochondrial membrane by one or two TMDs. On the other hand, Tob38 is likely a peripheral membrane protein that is strongly associated with Tob37 and other factors in the outer membrane.

### Role of transmembrane domains (TMDs) in Tob37

To assess the roles of the two possible TMDs of Tob37, we removed one or both of the domains from the protein coding sequence as shown in Figure 5A. Plasmid constructs encoding these mutant forms of Tob37 were used to transform the ΔTob37 sheltered heterokaryon. Histidine-requiring homokaryons expressing only the mutant forms of Tob37 were isolated, indicating that the ΔTob37 nucleus of the sheltered heterokaryon (Figure 1A) could be rescued by any of the three mutant versions of the protein. One strain from each transformation was chosen for further analysis: ΔTMD1-9 (lacking TMD1, Figure 5A), ΔCHD2-3 (lacking CHD), and ΔT+C12-5 (lacking both TMD1 and CHD). Mitochondria and post-mitochondrial pellet (referred to as cytosol) fractions were isolated from each strain. Western blot analysis showed that despite the removal of either or both of the domains, the shortened versions of Tob37 were targeted to, and remained associated with, mitochondria during standard isolation conditions while the control protein arginase [66], [67] was found predominantly in the cytosol fraction as predicted (Figure 5B). When mitochondria were treated with buffer containing 500 mM NaCl, all three mutant proteins still remained associated with the mitochondria (Figure 5C). Mitochondria isolated from the mutant strains were then subjected to alkali extraction with sodium carbonate at pH 11.0, 11.5, and 12.5 (Figure 5D). At pH 11.0, all versions of the protein remain in the membrane fraction. At pH 11.5, the wild type protein and the mutant lacking only the CHD were resistant to extraction from the membrane and remained in the pellet as did the control protein Tom40. However, the mutant proteins lacking either TMD1, or TMD1 and CHD were completely removed from the membrane by the alkali treatment. At pH 12.5, the behaviour of the protein lacking CHD is indistinguishable from the wild type protein. These data strongly suggest that TMD1 is a membrane spanning domain, but the CHD is not. This was further tested by examining the susceptibility of the mutant proteins to proteinase K treatment. If both TMD1 and CHD were membrane spanning domains, then loss of one of the domains could conceivably result in mislocalization of the large domain in the cytosol to the intermembrane space. However, all three mutant forms were shown to be digested when mitochondria were treated with proteinase K, as was the control protein Tom70. The intermembrane space control protein Tim13 was not digested. This suggests that no change in the location of the cytosolic domain resulted from deletion of either or both TMD1 and CHD (Figure 5E). This is consistent with the conclusion that only TMD1 spans the outer membrane and suggests that absence of TMD1 likely results in mislocalization of the small CHD to the cytosol (Figure 5F). Taken together, these data suggest that TMD1 serves as a membrane spanning tail-anchoring domain for *N. crassa* Tob37 (Figure 5F). The position of TMD1 within the protein is similar to the analogous region from the human Mtx1 protein (Figure 5G). In both proteins, the predicted TMD is flanked by regions containing positive charges, which is a characteristic of tail-anchoring mitochondrial sequences [38], [68], [69].

**Figure 6:**
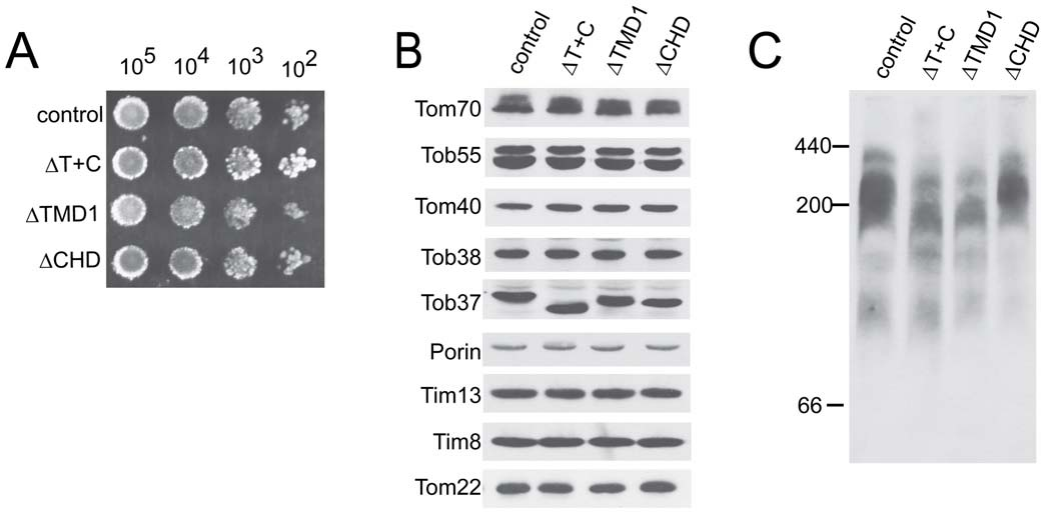
Characteristics of Tob37 C-terminal deletion strains. (a) Conidia from the strains indicated on the left were plated as described in the legend to Figure 1B. (b) Mitochondria were isolated from the strains indicated at the top of the panel and analyzed as described in the legend to Figure 1C. The control was strain 76–26. (c) Mitochondria isolated from the strains indicated at the top of the panel were dissolved in 1% digitonin, subjected to BNGE, transferred to PVDF, and decorated with antibody to Tob55. The position of molecular weight markers is indicated on the left.

The growth rates of strains expressing the mutant alleles of Tob37 were virtually identical to the control strain (Figure 6A) and there were no apparent alterations in the steady state levels of mitochondrial proteins (Figure 6B). However, lysis of isolated mitochondria with digitonin, followed by BNGE and western blot analysis revealed that the ratio of larger to smaller TOB complexes is somewhat reduced in mitochondria containing Tob37 proteins missing TMD1, or TMD1 plus CHD (Figure 6C). These data suggest that TMD1 of Tob37 plays a role in TOB complex assembly and/or stability.

**Figure 7:**
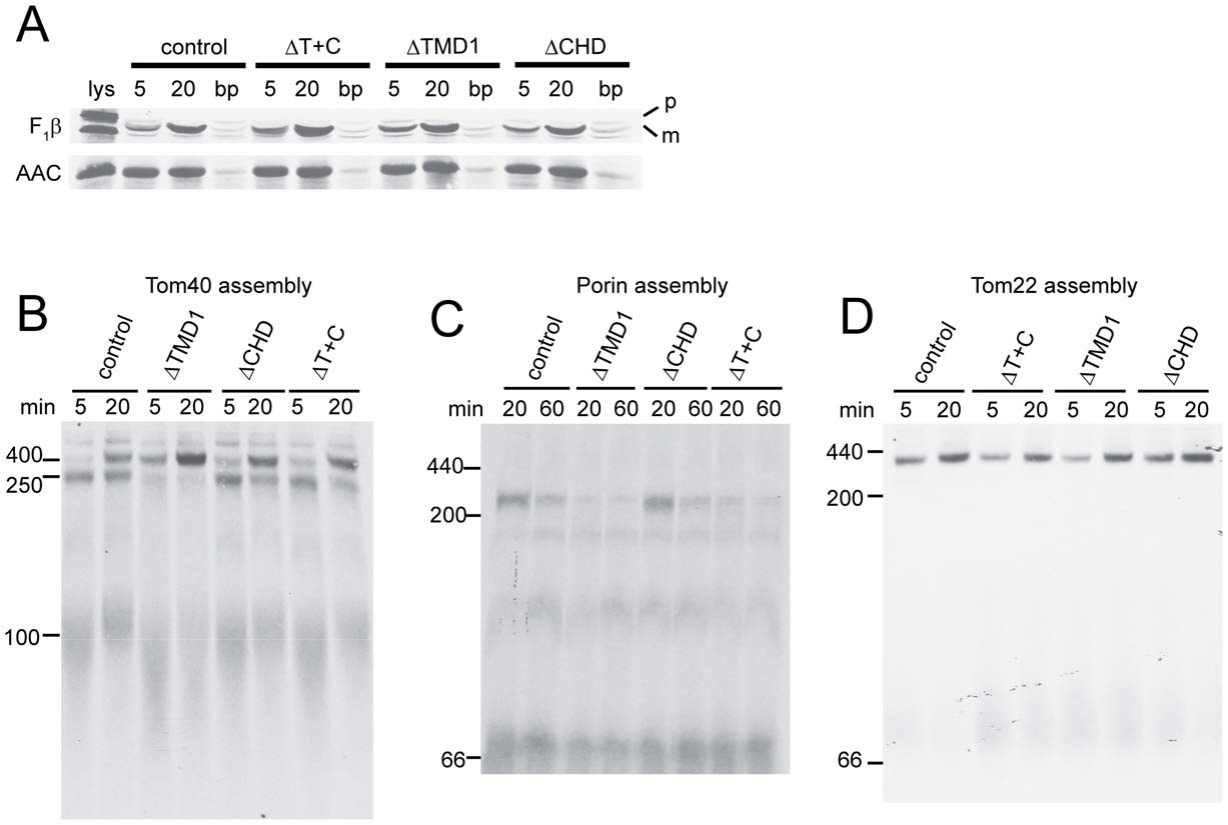
Role of Tob37 predicted TMDs on the import of mitochondrial precursor proteins into mitochondria. Import and assembly were analyzed as described in the legend to Figure 2 for the indicated precursors. (a) Import of F_1_β and AAC. (b) Assembly of Tom40. (c) Assembly of porin. (d) Assembly of Tom22.

We also investigated the import/assembly of mitochondrial proteins into mitochondria isolated from the strains containing the Tob37 mutant proteins. Import of F_1_β and AAC was indistinguishable from controls (Figure 7A). However, differences were observed with respect to the import of β-barrel proteins. For Tom40 (Figure 7B), mitochondria containing Tob37 lacking TMD1 assemble Tom40 into the final 400 kDa TOM complex at a slightly increased rate and accumulation of Tom40 precursor at the 250 kDa TOB complex intermediate stage is greatly reduced. However, mitochondria containing Tob37 lacking the CHD, or both TMD1 and the CHD, are only slightly different from the wild type control with respect to appearance of the precursor into the 250 kDa intermediate I and the final assembled complex. For porin (Figure 7C), mitochondria containing Tob37 lacking the CHD are indistinguishable from the wild type control. However, mitochondria with Tob37 lacking TMD1 do not efficiently form the highest molecular weight form (240 kDa). Other porin complexes are present at or near control levels. We have previously shown the 240 kDa form represents porin precursor bound to the TOB complex [27]. Assembly of the porin precursor in mitochondria lacking TMD1 plus the CHD resembles the pattern seen in those lacking only TMD1. Thus, the loss of TMD1 alone reduces the level of both Tom40 and porin precursors bound at the TOB complex. However, there is a clear difference in the assembly of the two precursors in the ΔT+C mitochondria. For Tom40 assembly is similar to the control, but for porin it is similar to the ΔTMD1 mitochondria. As shown in Figure 2E, the TOB complex is also required for the assembly of Tom22. Examination of Tom22 assembly into mitochondria with the TMD deletions revealed a slightly lower rate of assembly when TMD1 or TMD1 plus the CHD were deleted (Figure 7D), which is most clearly seen at the 5 min time point.

## Discussion

Despite the limited sequence similarity among the Tob37 and Tob38 proteins from different organisms (Figure S2), many of their characteristics and functions are conserved. *N. crassa* Tob37 and Tob38 are found in complexes with either Tob55 or with Tob55 and Mdm10, which are analogous to the core- and holo-TOB complexes, respectively, that have been described in *S. cerevisiae* [9], [10], [11], [12], [15], [35]. Tob38 is essential in both *N. crassa* and *S. cerevisiae* [9], [10], [11]. Its function in β-barrel import is common to these two organisms as well as mammals [40], as is its topology as an alkali extractable peripheral membrane protein. Tob37 is an essential protein in *N. crassa* and is required for embryonic development in mice [43]. Although the *S. cerevisiae* protein is not essential, knockouts have growth defects at high temperatures [30]. Loss of Tob37 in *C. albicans* leads to a much reduced growth rate [36]. Tob37 is involved in β-barrel protein assembly in *S. cerevisiae* [32] and *N. crassa*. This has not been shown directly in mammals, but Mtx1 is known to associate with Mtx2 and its levels decrease in Mtx2 knockdowns suggesting a functional relationship between the two proteins [39], [40]. The *N. crassa* and mammalian Tob37 proteins both contain a tail-anchoring domain. Although potential TMDs were detected in the *S. cerevisiae* protein [30], it was concluded that it is a peripheral membrane protein based on alkaline extractability [31]. In mammals deletion of the anchoring domain has a severe effect on targeting Mtx1 to mitochondria [38]. Deletion of TMD1 in the *N. crassa* protein results in loss of resistance to alkaline extraction. However, when TMD1 is absent, the *N. crassa* protein still associates with mitochondria. Thus, *N. crassa* Tob37 seems to possess a mixture of the properties found in the *S. cerevisiae* and mammalian proteins. The finding that mammalian Mtx1 and Mtx2 interact led to the suggestion that Mtx2 is bound to the mitochondrial membrane by Mtx1 [39]. Similarly, one of the suggested roles of *S. cerevisiae* Tob37 is to stabilize the Tob38 protein or the TOB complex in general [33], [34]. Our finding that Tob38 is greatly reduced in the Tob37 knockout, but Tob37 is only slightly reduced in the Tob38 knockout agrees with these suggestions. However, our results also show that when *N. crassa* Tob37 levels are reduced to virtually undetectable levels, about half of the Tob38 that associates with mitochondria is still resistant to alkali extraction, suggesting that the protein also binds tightly to other components of the outer membrane, possibly by a specific interaction with Tob55 [28]. Our observation of the three Tob proteins in complexes with or without Mdm10 is in agreement with findings in *S. cerevisiae*. However, we also detected additional complexes that appear to contain only Tob55. The physiological relevance and role of these complexes remains to be determined.

Import of radiolabeled Tom40 precursor into ΔTob37 or ΔTob38 mitochondria was reduced. Some accumulation into the fully assembled complex was observed, but no precursor was detected in an intermediate that would represent a lower molecular weight version of the TOB complex that lacked a core subunit. These results differ from those observed in previous studies of Tob37 deficient mitochondria of *S. cerevisiae* where accumulation of the Tom40 precursor was observed in a lower molecular weight TOB complex lacking Tob37 with only very small amounts of Tom40 reaching the final assembled state [12], [32], [34]. On the other hand, our finding that some Tom40 does reach the assembled complex is similar to a more recent study in *S. cerevisiae* where substantial assembly was observed after 60 min of import in mitochondria lacking Tob37 [70]. Our results for Tom40 assembly into mitochondria deficient in Tob38 are similar to one previous study in *S. cerevisiae* that used mitochondria with reduced levels of Tob38. In that report, Tom40 precursor reached the fully assembled TOM complex in a time-dependent manner, in amounts similar to controls, with no accumulation at lower molecular weight intermediates—though it was also shown that a proportion of the protein was not properly assembled [9]. In the present study we observed somewhat reduced levels of Tom40 reaching the assembled TOM complex. Alkali extraction and protease susceptibility studies demonstrated that most, if not all, of that protein was properly assembled into the membrane. Curiously, compared to our findings and the aforementioned yeast work, others have reported quite different results for the effects of Tob38 depletion in *S. cerevisiae*. In one study, virtually no Tom40 precursor reached the final TOM complex and the amount of the precursor accumulated at intermediate I was reduced [11]. Similar results were seen using a temperature-sensitive allele of Tob38 [10]. Whether these differences reflect minor alterations in the mitochondria resulting from experimental approaches, or differences between organisms or strains, remains to be determined.

The assembly of porin was also reduced in mitochondria deficient in Tob37 or Tob38. These results are similar to findings with *S. cerevisiae* cells deficient in Tob37 [32], [34] or Tob38 [9], [10], [11] though the effects in *N. crassa* appear to be more dramatic. Mammalian mitochondria depleted of Mtx2 also show decreased assembly of both porin and Tom40 [40]. Our assembly assays for Tom40 or porin in Tob37 or Tob38 deficient mitochondria show much reduced or undetectable levels of precursors bound at the TOB complex. Two explanations for similar observations have been given previously [34], [35]. Deficiency of the proteins may influence the efficiency of binding precursors to the complex. Alternatively, delays in processing precursor bound to the complex, might make them susceptible to increased degradation. Interestingly, when ΔTob37 mitochondria are used in our Tom40 assembly assays, there is accumulation of labelled precursor in a smear at the position of the 100 kDa intermediate II. In the ΔTob38 mitochondria there is virtually no material seen in this region. This suggests that precursor may be less efficiently integrated into the membrane when Tob38 is absent. When Tob37 is deficient, the Tom40 precursor appears to enter the membrane but may not be properly assembled with other Tom subunits to give a discrete 100 kDa form. If true, this observation would lend support to an earlier suggestion that intermediate II may still be associated with the TOB complex [33].

We observed a slight reduction in the import of the matrix targeted F1β and the inner membrane protein AAC in mitochondria deficient in Tob37 and Tob38. Various results have been reported for the effects of deficiencies of Tob37, Tob38, or metaxins on the import of matrix and inner membrane precursors into mitochondria. In some studies the import of at least some of these proteins is reduced [30], [34], [38], [41], in others it is not [9], [10], [11], [32], [33], [40]. One explanation might be that different strategies used to eliminate or reduce Tob37 and Tob38 levels or activity result in variations of the steady state levels of other proteins required for import of proteins to other subcompartments. In fact, one study has shown that increasing TOM complex stability by overexpressing Tom6 improved the import of matrix precursor proteins in Tob37 deficient mitochondria [34].

The steady state levels of various proteins in ΔTob37 and ΔTob38 mitochondria were reduced, most notably Tom5 and Mdm10. Thus, it might be argued that the defects in β-barrel assembly and the slightly decreased import of F_1_β and AAC observed in our experiments and at least some other previous studies, are due to reduced levels of other mitochondrial proteins that are involved in import or assembly. However, for the β-barrel precursors this seems unlikely for the following reasons. The non-core TOB complex proteins most likely to have an effect on β-barrel assembly would be TOM complex components, the small Tim proteins, and Mdm10. The patterns of assembly for Tom40 and porin in ΔTob37 and ΔTob38 mitochondria do not resemble those observed when the small Tim proteins [5] (Figure S1), the small Tom proteins [71], or Mdm10 [16] are depleted. On the other hand, the assembly patterns observed in the present study are similar to those observed when Tob55 levels are depleted [27]. Furthermore, the phenotypes observed for Tom40 and porin assembly are very similar in ΔTob37 mitochondria and ΔTMD1 mitochondria, which would argue that a similar process is affected in both cases. In ΔTMD1 mitochondria import of F_1_β and AAC is not affected and no changes in the steady state levels of other mitochondrial proteins were observed, supporting the notion that alterations in the Tob proteins are responsible for the effects on β-barrel assembly.

**Figure 8:**
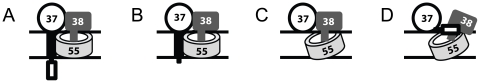
Hypothetical model for effects of Tob37 alterations on the TOB complex. Tob55 is shown as a pore-containing light grey ring embedded in the membrane. Tob37, is represented as in Fig. 5F with TMD1 and CHD in the outer membrane and intermembrane space, respectively. Tob38 is shown in dark grey with a domain extending into the pore of Tob55. A. The normal TOB complex. B. CHD absent. Loss of CHD has no effect on porin assembly and mild effects on Tom40 assembly. All members of the complex are shown in their normal configuration C. TMD1 and CHD absent. This results in reduced accumulation of the porin precursor at the TOB complex. Effects on Tom40 are mild. A conformational change in Tob55 is shown as one possible effect caused by loss of TMD1 resulting in porin assembly defects. D. Only TMD1 absent. This results in reduced accumulation of both porin and Tom40 precursors at the TOB complex. The conformational change in Tob55 due to lack of TMD1 is shown as in C. However, an additional change due to the suggested mislocalization of the CHD, is represented as an effect on Tob38.

Our studies on assembly of β-barrel proteins in mitochondria bearing Tob37 proteins with C-terminal alterations have shown that loss of the CHD has virtually no effect. However, the accumulation of the precursors for both Tom40 and porin, at the stage of interaction with the TOB complex, is severely reduced when TMD1 is removed. Interestingly, simultaneous removal of both of these domains has a different effect on the two β-barrel precursors examined. For Tom40, removal of both domains restores both the accumulation of the precursor with the TOB complex and its assembly into the TOM complex to near wild type levels. These data suggest that it is not the absence of TMD1 that affects Tom40 assembly, but the predicted mislocalization of the CHD. In a Tob37 protein lacking the tail-anchoring TMD1, the CHD would be expected to be found on the outer surface of the outer membrane, rather than in the intermembrane space (Figure 5F). We speculate that the mislocalized CHD alters interactions between TOB complex components such that binding of the Tom40 precursor is affected. The more rapid assembly of the precursor to the final complex suggests that when TMD1 alone is absent, the precursor is released from the TOB complex more quickly. This would be in keeping with a role for Tob37 in precursor release as suggested in two recent studies of the *S. cerevisiae* protein [33], [34]. The more rapid release in the mutant may suggest that binding at the precursor stage may represent a quality control stage for Tom40 assembly, allowing it to achieve proper conformation and/or combining with other subunits. However, for the precursor of porin, removal of both domains gives an assembly phenotype similar to missing only TMD1. This suggests that the effects of CHD mislocalization do not have the same effect on the porin precursor. Thus, individual β-barrel precursors may associate with different features of the TOB complex to achieve maximal productive interactions. Alternatively, the nature of TOB complex/precursor interactions may specifically affect downstream assembly steps that differ between the precurors. One of many possible models to account for these observations is given in Figure 8. Continued investigations will be required to reach a fuller understanding of Tob37 topology and its relationship to TOB complex structure and function.

## Supporting Information

Figure S1 **Controls for effect of damaged outer membranes in isolated mitochondria from mutant strains on mitochondrial protein import/assembly.** Mitochondria from control strain HP1 grown in the presence of histidine and fpa were subjected to brief periods of vortexing in the presence of swelling buffer to produce mitochondria with damaged outer membranes as described previously [16]. These mitochondria were then compared to undamaged control mitochondria and mitochondria from strains ΔTob37 and ΔTob38 grown in the presence of histidine and fpa to reduce levels of Tob37 and Tob38. Import and assembly assays were as described in the legend to Fig. 2. (A) Assembly of Tom40. (B) Assembly of porin. (C) Import of F_1_β and AAC. (D) Assembly of Tom22.
(PDF)

Figure S2 **Alignments of** ***Neurospora crassa*** **(Nc),** ***Saccharomyces cerevisiae*** **(Sc), and** ***Homo sapiens*** **(Hs) Tob37 and Tob38 proteins.** *, identical residues; :, conserved substitutions; ., semi-conserved substitutions. For the Tob37 alignment, the yellow highlight shows TMD1 in the *N. crassa* protein and the TMD of the *H. sapiens* protein, and the blue highlight shows the CHD of the *N. crassa* protein.
(DOCX)
