# Supplementary figures and images for: The Neurospora crassa TOB Complex: Analysis of the Topology and Function of Tob38 and Tob37
Source: PLoS One. 2011 Sep 28;6(9):e25650. doi: 10.1371/journal.pone.0025650 (PMC3182244; doi:10.1371/journal.pone.0025650)

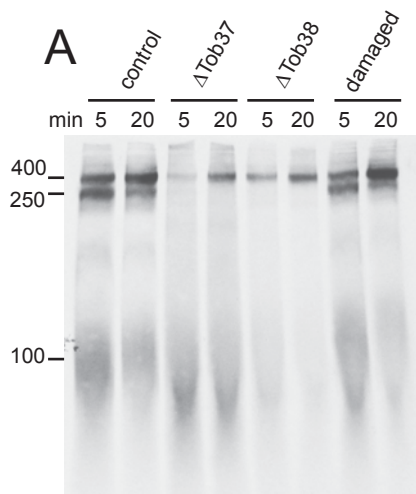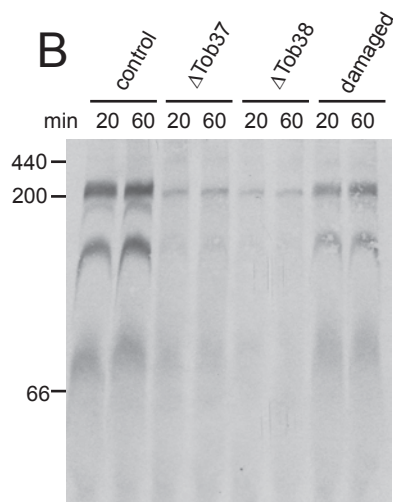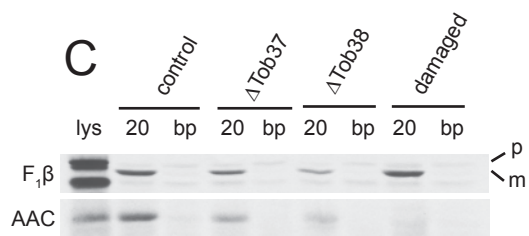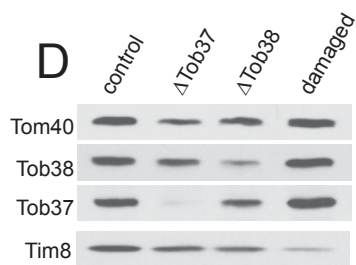

Supplement: Figure S1 — Controls for effect of damaged outer membranes in isolated mitochondria from mutant strains on mitochondrial protein import/assembly. Mitochondria from control strain HP1 grown in the presence of histidine and fpa were subjected to brief periods of vortexing in the presence of swelling buffer to produce mitochondria with damaged outer membranes as described previously [16]. These mitochondria were then compared to undamaged control mitochondria and mitochondria from strains ΔTob37 and ΔTob38 grown in the presence of histidine and fpa to reduce levels of Tob37 and Tob38. Import and assembly assays were as described in the legend to Fig. 2. (A) Assembly of Tom40. (B) Assembly of porin. (C) Import of F1β and AAC. (D) Assembly of Tom22. (PDF) [file pone.0025650.s001.pdf]
