# Supplementary material for: The Neurospora crassa TOB Complex: Analysis of the Topology and Function of Tob38 and Tob37
Source: PLoS One. 2011 Sep 28;6(9):e25650. doi: 10.1371/journal.pone.0025650 (PMC3182244; doi:10.1371/journal.pone.0025650)
Supplement: Figure S2 — Alignments of Neurospora crassa (Nc), Saccharomyces cerevisiae (Sc), and Homo sapiens (Hs) Tob37 and Tob38 proteins. *, identical residues; :, conserved substitutions; ., semi-conserved substitutions. For the Tob37 alignment, the yellow highlight shows TMD1 in the N. crassa protein and the TMD of the H. sapiens protein, and the blue highlight shows the CHD of the N. crassa protein. (DOCX) [file pone.0025650.s002.docx]

**Tob37 alignment**

***Nc* --MTLELHVWGPAFGLPSIDAECLATVTYFAQTLSAADYLLVQSSPSAVPS-------HH 51**

***Hs* MAAPMELFCWSGGWGLPSVDLDSLAVLTYARFTGAPLKVHKISN-PWQSPS-------GT 52**

***Sc* -MVKGSVHLWGKDGKASLISVDSIALVWFIKLCTSEEAKSMVAGLQIVFSNNTDLSSDGK 59**

**.:. *. . :. :.:* : : : : . ..**

***Nc* LPALYNPSTATWISGFDPIVNYLSTLQPP------SYHHPDVTTLPSRVYADSQAYKALL 105**

***Hs* LPALR-TSHGEVISVPHKIITHLRKEK-----------YNADYDLSARQGADTLAFMSLL 100**

***Sc* LPVLI-LDNGTKVSGYVNIVQFLHKNICTSKYEKGTDYEEDLAIVRKKDRLLEYSLLNYV 118**

****.* . . :* *: .* . : : : :**

***Nc* TSSAAPLLALSLYVSSANYSETTRPAYSAILPFPLPWTEPLAVRAAMAARAAHLGMSSLD 165**

***Hs* EEKLLPVLVHTFWIDTKNYVEVTRKWYAEAMPFPLNFFLP-------------------- 140**

***Sc* DVEISRLTDYQLFLNTKNYNEYTKKLFSKLLYFPMWYNTP-------------------- 158**

**. : :::.: ** * *: :: : **: : ***

***Nc* TDAEMERLEREEREREAAGWVQIPKALRKAVGGQNSGVKGQLSPEMKRRIKLEGLAAEVF 225**

***Hs* -----GRMQRQYMER-----------LQLLTGEHRPEDEEELEKELYR--EARECLTLLS 182**

***Sc* -----LQLRSQAREN-----------CEEIIGSLTLEDDEEFVESKAMESASQLAQSKTF 202**

**::. : *. . * . :: . . :**

***Nc* DVLGEVDFLEEEDGEEEEEEEEEAKEGGARIKVTLETKCLAFAYLALMLLPEVPRPWLKE 285**

***Hs* QRLGSQKFFFG--------------------DAPASLDAFVFSYLALLLQAKLPSGKLQV 222**

***Sc* KIAHKNKIKGK-----------------------QELQQVKYNLQFDNRLQSCVSNWLAA 239**

**. . .: . . . : . ***

***Nc* VLQKKYAGLCKFVLEYRRKTFPDSGKVLPWADRESDPAVSACDSALSIVGRFVRAVIDDI 345**

***Hs* HLRGLHN-LCAYCTHILSLYFPWDGAEVPPQ-RQTPAGPETEEEPYRRRNQILSVLAGLA 280**

***Sc* RKKLDDSVILSSDLLFLANLYVQLGLPDGNRIRSKLEQTFGSELLNSMSNKIDDFVHRPS 299**

**: : : * *.. : .:: :**

***Nc* PMLGREWSRWWALRQRRVAEENSAETQLVVRRSVGESERSLLLAGAGLTLLAINVAGLGI 405**

***Hs* AMVG------YALLSGIVSIQRATPARAPGTRTLGMAEEDEEE----------------- 317**

***Sc* NNLE----------------QRDPQFREQGNVVMSLYNLACKYI---------------- 327**

**: :. . : :. :**

***Nc* YWYRYRGLLGAPLQTWHRPLVGLGSFGAAGAMFAGLA 442**

***Hs* -------------------------------------**

***Sc* -------------------------------------**

**Tob38 alignment**

***Nc* MATTSAAAPPRKWWQVPRPLQKVFDTFPLLAYDVNALPARAQSATSGDLPTLYVFSTEEE 60**

***Hs* -------------------MSLVAEAFVSQIAAAEPWPENAT---------LYQQLKGEQ 32**

***Sc* MVSS---------FSVPMPVKRIFDTFPLQTYAAQTDKDEAVALEIQRRSYTFTERGGGS 51**

**:. : ::* .:. .* : .**

***Nc* ALLGAPSFNPNCLKWQAFLKLAGVKFQILP--STNHASPTGALPFILPTRSSPTDAPSPI 118**

***Hs* ILLSD---NAASLAVQAFLQMCNLPIKVVCRANAEYMSPSGKVPFIHVGNQVVSELGPIV 89**

***Sc* SDLTVEGTYKLGVYNVFLEANTGAALATDPWCLFVQLALCQKNGLVLPTHSQEQTPSHTC 111**

*** : : . : : :: ..**

***Nc* PSSKLHDYALKYGTSNPPEVSALRLDAYQA-LLDVPIRNAWLQALYRDPEYTDLLDRFYI 177**

***Hs* QFVKAKGHSLSDG---LEEVQKAEMKAYME-LVNNMLLTAELYLQWCDEATVGEITHARY 145**

***Sc* N----HEMLVLSRLSNPDEALPILVEGYKKRIIRSTVAISEIMRSRILDDAEQLMYHTLL 167**

**: *. :..* :: : : : : :**

***Nc* TPASSSYWVRGALRHQLRRAAETEILKTGPGGAASTAVSLLVDEHSVYRAAVQALEALAT 237**

***Hs* G-SPYPWPLNHILAYQKQWEVKRKMKAIGWG--KKTLDQVLEDVDQCCQALSQRLGTQP- 201**

***Sc* DTVLYDCWITQILFCASDAQFMELYSCQKLSDSIVTPLDVENSLLQKLSAKSLKISLTKR 227**

**: * . * .: . . * :**

***Nc* LLSESKTGWFFGAETPTIFDASVFAYTHLMLKYMSDAEGEVEGNMGFILASRKLGTMVRS 297**

***Hs* ---------YFFNKQPTELDALVFGHLYTILTTQ--------------LTNDELSEKVKN 238**

***Sc* NKFQFRHREIVKSMQGVYHNHHNSVNQEQVLNVLFENSKQVLLGLKDMLKSDGQPTYLHL 287**

**. . : :*. * . ::**

***Nc* AGSGELEQHHRRLFELLWLADSNAELLDAKARGNKLLQFQLQA 340**

***Hs* YSN--LLAFCRRIEQHYFEDRGKGRLS---------------- 263**

***Sc* KIASYILCITNVKEPIKLKTFVENECKELVQFAQDTLKNFVQ- 329**
